# Supplementary material for: Crystal structure of undecaprenyl-pyrophosphate phosphatase and its role in peptidoglycan biosynthesis
Source: Nat Commun. 2018 Mar 14;9:1078. doi: 10.1038/s41467-018-03477-5 (PMC5852022; doi:10.1038/s41467-018-03477-5)
Supplement: Supplementary file 1 — Supplementary Information [file 41467_2018_3477_MOESM1_ESM.pdf]

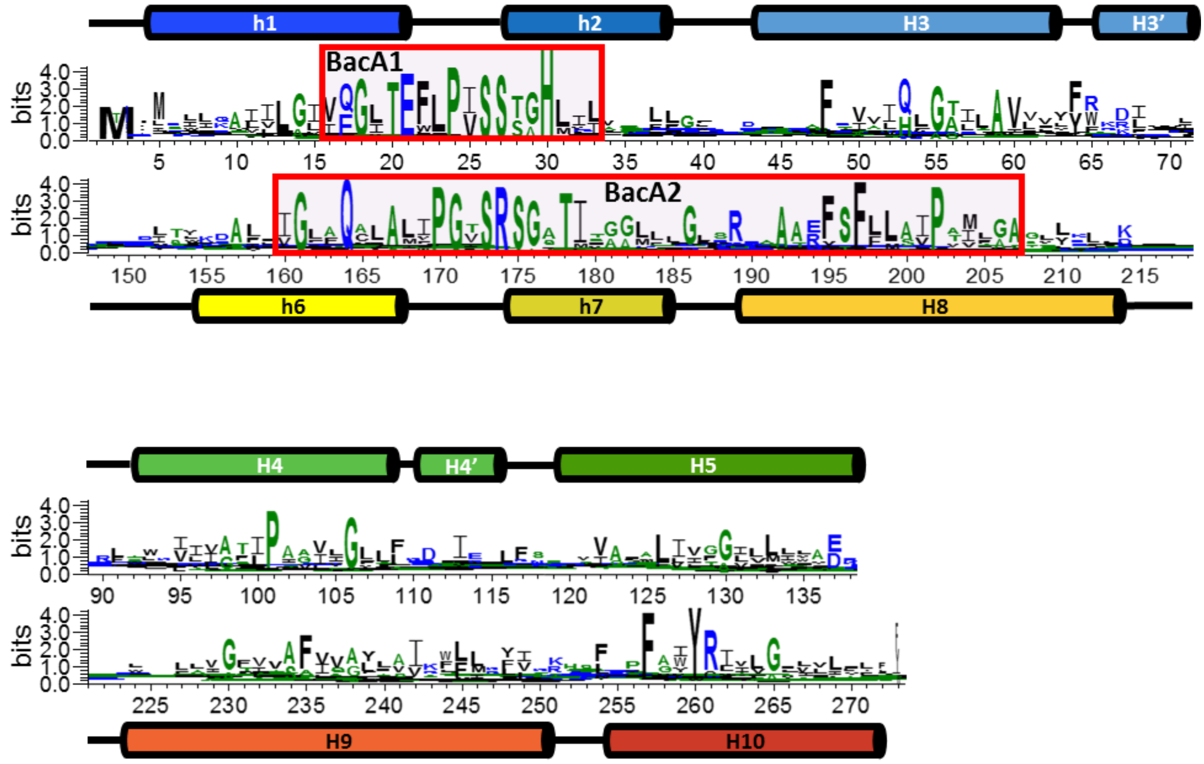

Supplementary Figure 1 | Sequence and secondary structure conservation comparisons in the N- and C-terminal repeats of BacA. A sequence Weblogo was constructed using 5,564 non-identical BacA sequences derived from the UniProKB database[1]. The conserved sequence motifs BacA1 (residues 16-33) in the N-terminal repeat and BacA2 (residues 160-207) in the C-terminal repeat are indicated by red boxes. Secondary structure is shown above and below the aligned sequences of the N- and C-terminal repeats, respectively. The majority of the high sequence conservation is contained within the two repeats of the two re-entrant helices h1-h2 and h6-h7. Residue numbering is taken from *E. coli*.

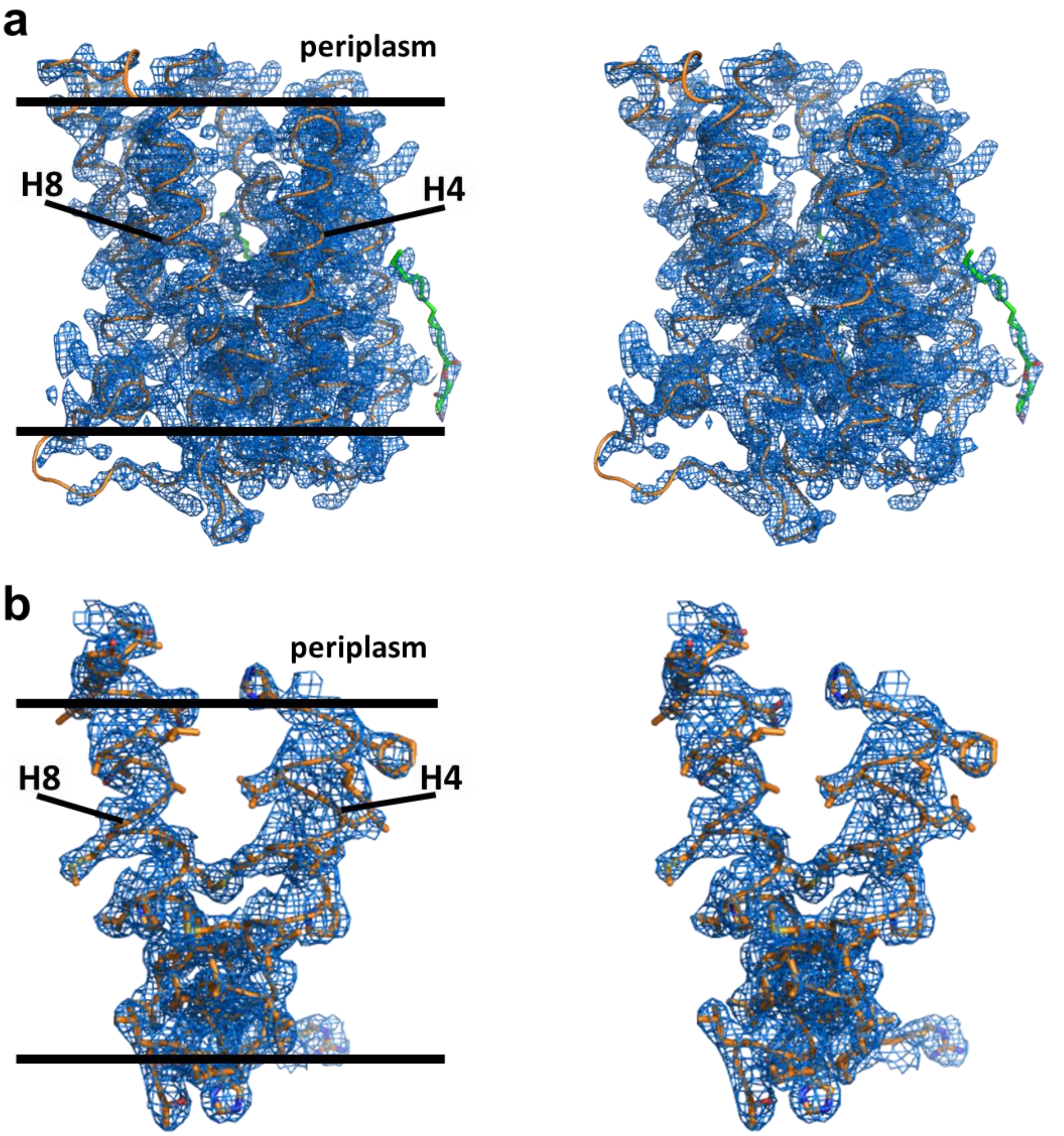

Supplementary Figure 2 | Stereo images of BacA. (**a**) 2Fo-Fc map of BacA. Backbone in cartoon representation (orange) with side chains omitted and contoured at 1.5 σ (blue mesh). Monoolein in stick representation (green). (**b**) 2Fo-Fc map of H4 and H8 in BacA. Backbone in cartoon representation with side chains visible (orange) and contoured at 1.0 σ (blue mesh).

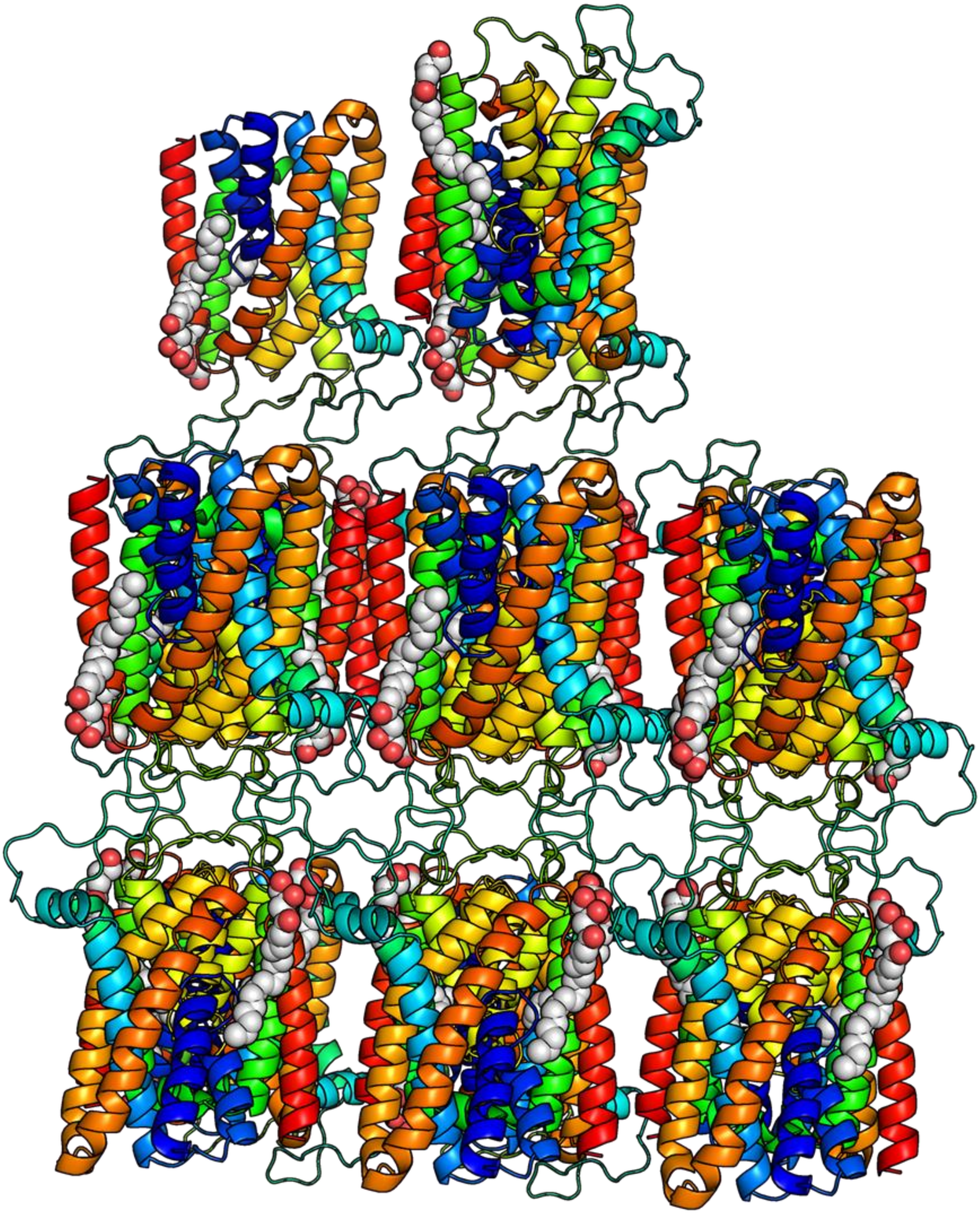

Supplementary Figure 3 | Type I crystal packing. BacA crystal packing is of the layered Type I variety. Type I packing is a hallmark of membrane protein crystals grown by the *in meso* method. BacA is shown in cartoon representation and rainbow colored from N- to C-terminus. Lipid (monoolein) molecules are shown as spheres with carbon and oxygen atoms colored white and red, respectively. Within layers, crystal contacts include protein-protein and protein-lipid interactions. Between layers, contacts are strictly of the protein-protein type.

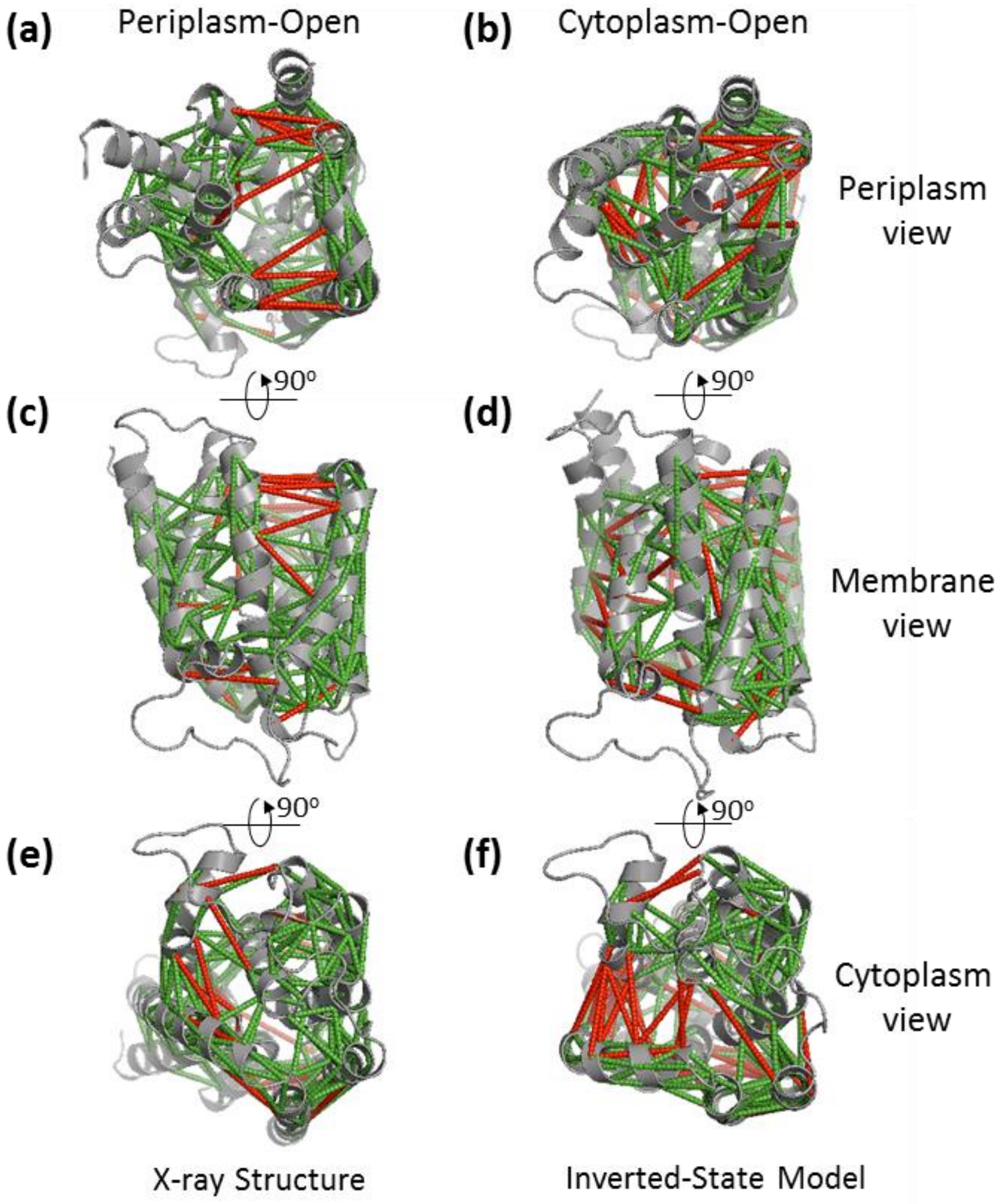

Supplementary Figure 4 | Co-evolutionary analysis. Predicted residue contact pairs mapped onto the (**a, c, e**) X-ray structure and (**b, d, f**) the inverted-state model of BacA. Residue pairs were predicted through co-evolution analysis of the 14,369 non-identical metagenomics sequences homologous to BacA. Pairs of residues that have a C_α distance of less than 10 Å are identified by a green connecting line. Predicted contacts that are greater than the 10 Å threshold are joined by a red line. Interestingly, the predicted contacts longer than 10 Å in the crystal structure, in particular in (**a**) and (**c**), indicate the potential conformational closure of both the periplasmic opening and membrane fenestration.

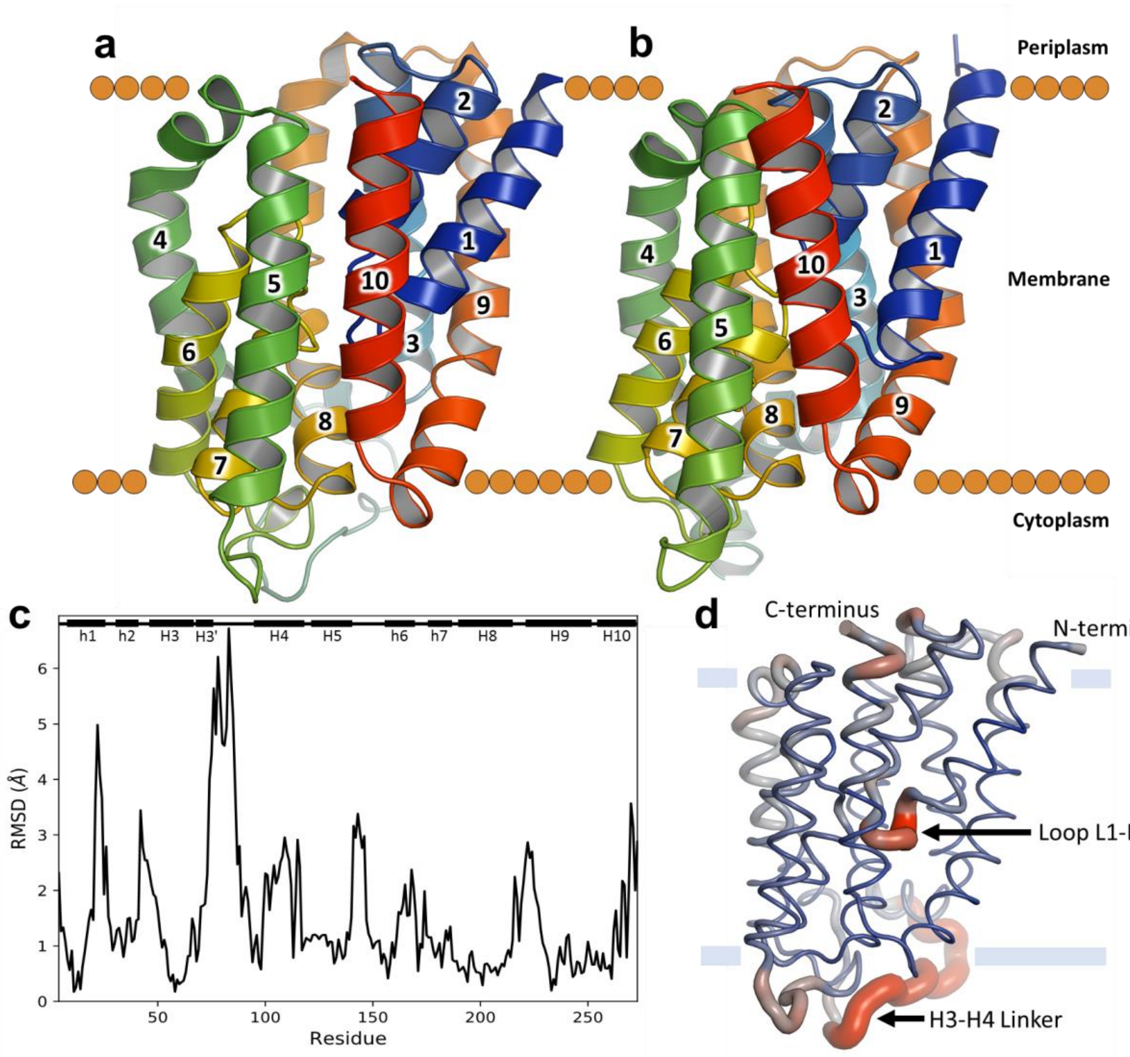

Supplementary Figure 5 | Comparison between the X-ray crystal and predicted structures of BacA. (**a**) Crystal structure of BacA. (**b**) The co-evolution-derived molecular model of BacA. The Cα r.m.s.d between the two structures is 3.79 Å over 270 residues. (**c**) C_α r.m.s.d between the X-ray structure and the Rosetta model of BacA[2] shown per amino acid residue. (**d**) The C_α-r.m.s.d values illustrated on a putty cartoon representation of the X-ray structure of BacA, on a blue-white-red (thin to thick) scale, where a blue and thin cartoon depicts low fluctuation, while a red and thick cartoon shows high deviation from the X-ray structure. The regions of greatest difference are in the hair-pin loop between h1 and h2 (Loop L1-L2) and the linker connecting H3 and H4.

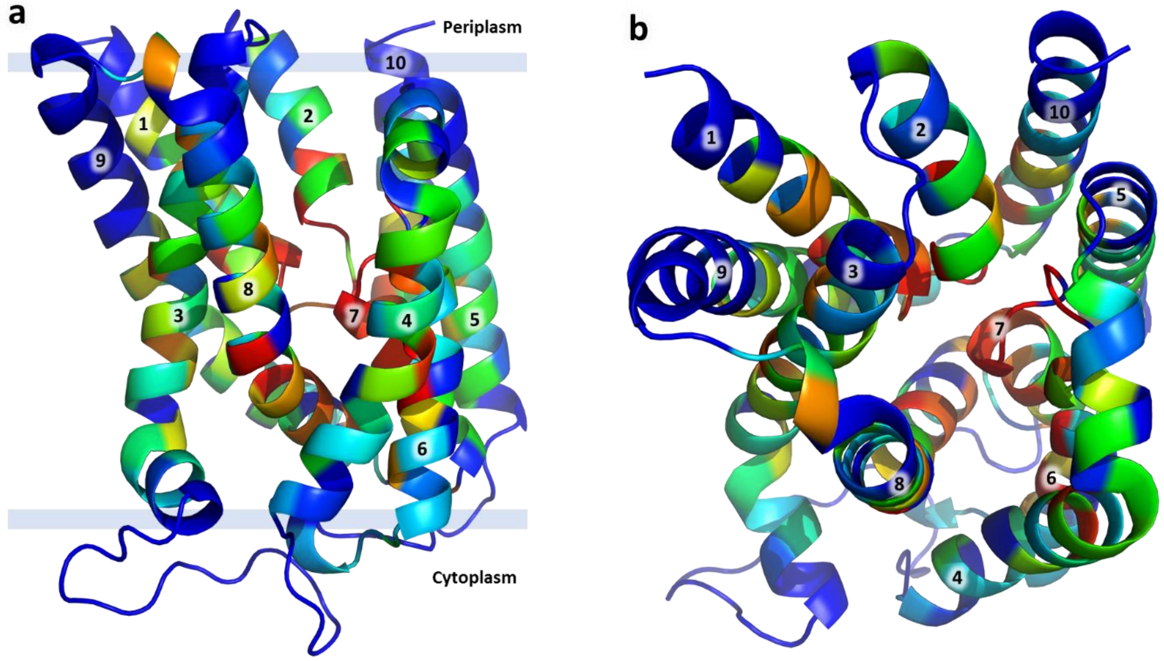

Supplementary Figure 6 | The putative active site of BacA. (**a**) Residue conservation derived from the BacA metagenomics sequences mapped onto the structure B-factor. Conservation is highlighted on a blue to red scale, from weak to high conservation. The view is from the membrane through the cleft between H4 and H8. (**b**) A view from the periplasm into the active site.

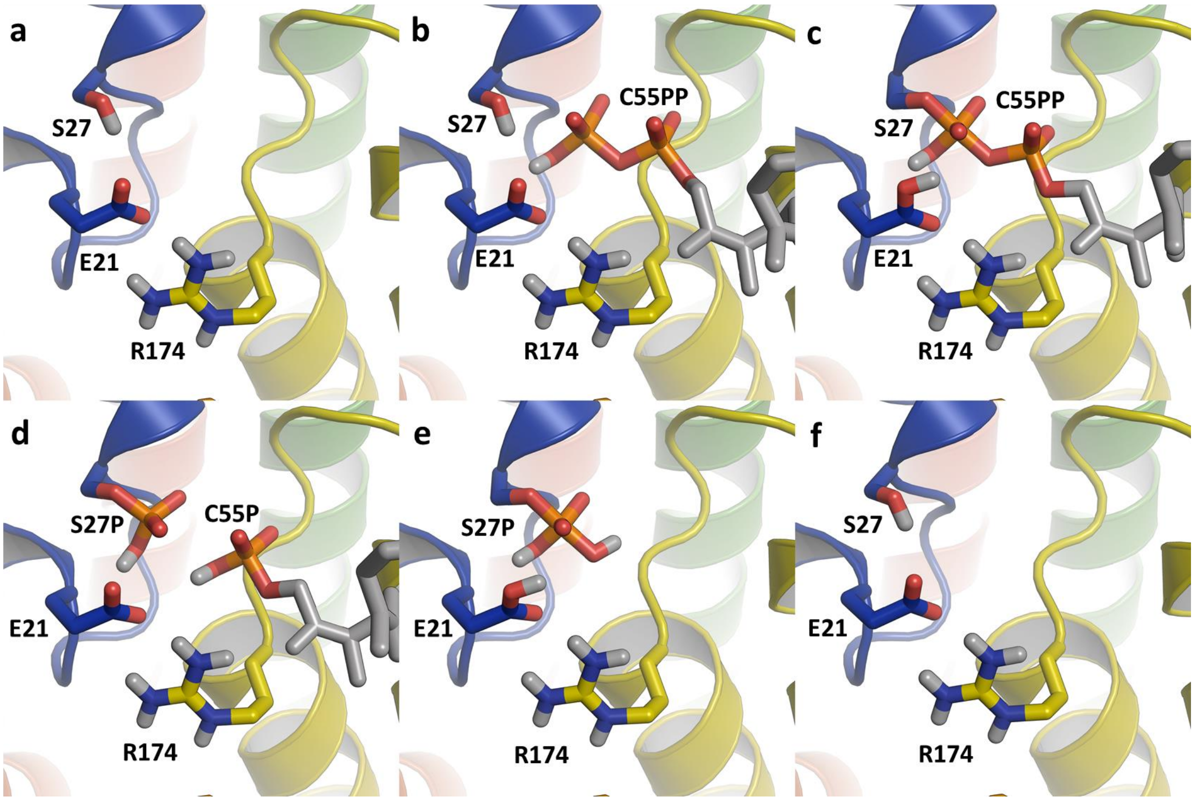

Supplementary Figure 7 | Steps in the undecaprenyl-pyrophosphate phosphatase reaction as visualized by MDS. (**a**) Resting state, with Ser27 protonated, Glu21 deprotonated and Arg174. (**b**) Binding of C55PP to the active site. (**c**) Formation of the pentavalent C55PP intermediate stabilized by Arg174. The proton on Ser27 has transferred to Glu21. (**d**) Reaction product C55P with phosphorylated Ser27. In the MDS there are a number of water molecules in the vicinity of phosphorylated Ser27 that may participate in the hydrolysis reaction. (**e**) The pentavalent phosphorylated Ser27 intermediate and protonated Glu21 have formed following the hydrolysis reaction. C55P has left the binding site possibly aided by Arg174. (**f**) Following collapse of the pentavalent intermediate the phosphate has exited the binding pocket restoring the active site to its starting condition as in (**a**). Each state has been simulated in MDS, with the protein and ligands stable as shown. See also **Supplementary Movie 1.**

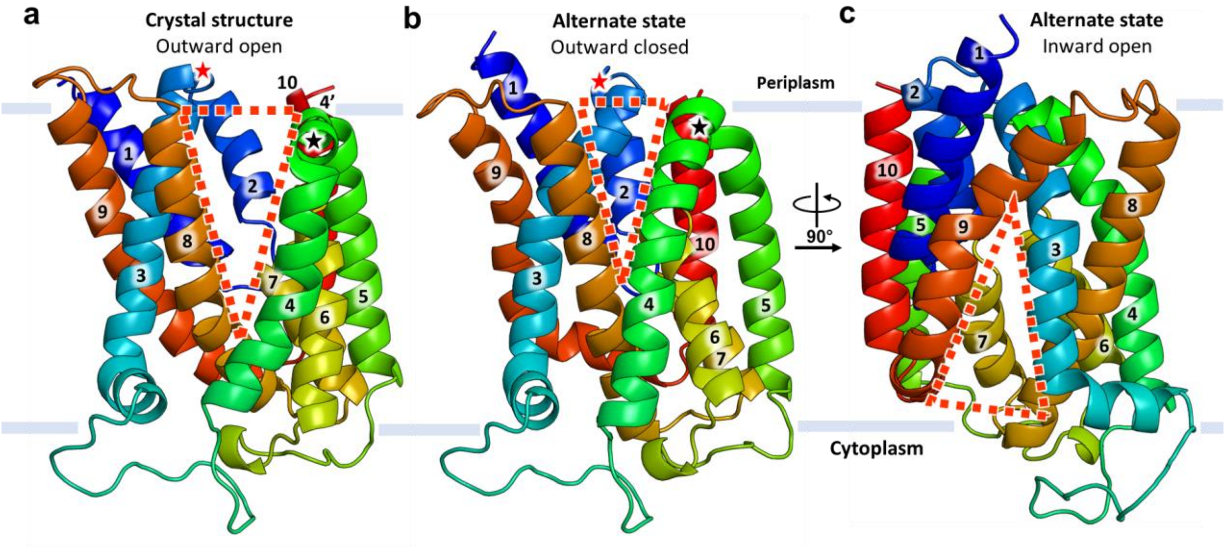

Supplementary Figure 8 | View from the membrane into the open pocket of BacA in its crystal and alternate states. (**a**) Crystal structure, open to the periplasm. The cleft (dashed triangle) between the pocket and the membrane lies between H4 and H8. Residues mutated to cysteine to evaluate structural changes during catalysis include Gly42 on loop L2-3 (red star) and Phe117 on H4' (black star). (**b**) Model alternate state. The pocket is closed to the periplasm. (**c**) Model alternate state, open to the cytoplasm. The cleft between the pocket and the membrane lies between H3 and H9 in the alternate state.

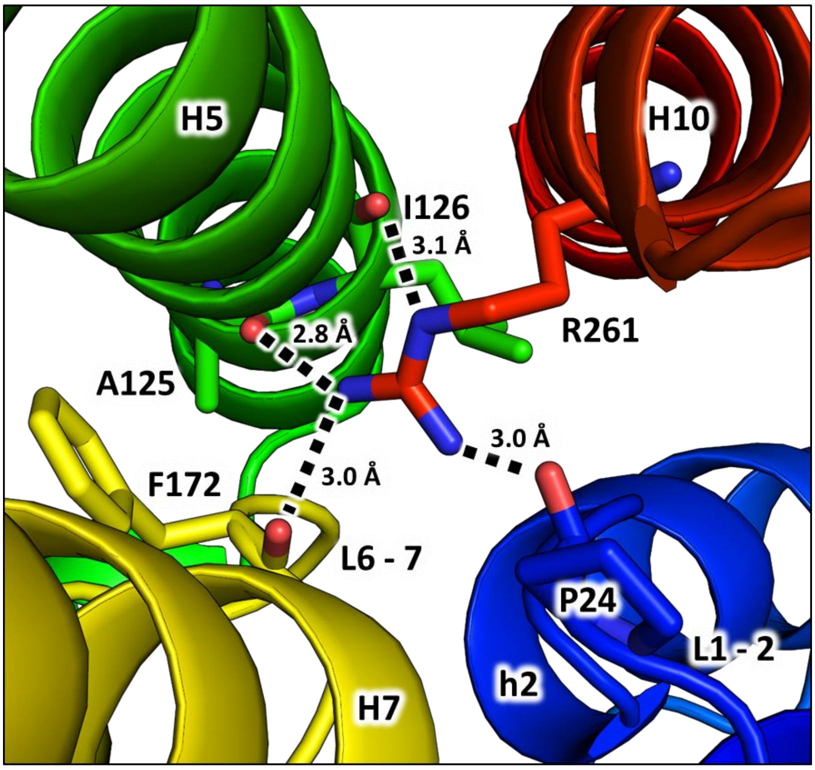

Supplementary Figure 9 | Arg261 locks the two domains together. The guanidinium of Arg261 in H10 (domain 1) interacts with carbonyl oxygens in H5 (Ala125, Ile126; domain 2), loop L1-2 (Pro24; domain 1) and loop L6-7 (Phe172; domain 2) to bind the two domains together. Arg261 is strictly conserved and essential for function. In addition to holding the domains together, Arg261 likely plays an important role in stabilizing a functional active site by interacting with both re-entrant loops.

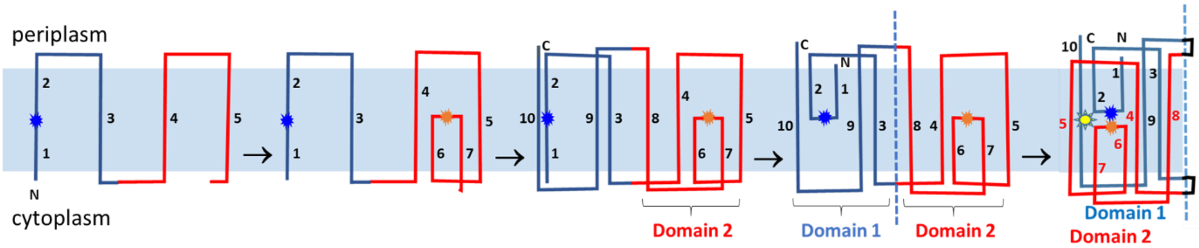

Supplementary Figure 10 | BacA synthesis and folding in the membrane. The proposed mechanism advances from left to right. Helices are numbered and re-entrant loops identified by star bursts (blue, tan). The light blue background represents the membrane. Rotation axis about which final folding occurs to bring the two domains together is indicated by the dashed line. Domain 2 consists of contiguous helices and is the first of the two domains to form. As soon as the synthesis of the components of domain 1 is completed and domain 1 assembles it folds over to pair with domain 2 creating an enclosed active site where the two re-entrant loops come together toward the bilayer mid-plane. The paired domains are stabilized by Arg261 (yellow star) on H10 engaging with backbone carbonyls in H5 and the re-entrant loops (**Supplementary Fig. 9**).

Supplementary Table 1 | Specific activity of BacA constructs as polyisoprenyl-pyrophosphate phosphatases.

Construct	Relative Specific Activity*	Reference
Gly42Cys/Phe117Cys (- βME)	2	This work
Gly42Cys/Phe117Cys (+ βME)	71	This work
Glu17Ala/Glu21Ala	<1	Chang *et al.*, 2014
His30Ala	<1	Chang *et al.*, 2014
Ser173Ala	<1	Chang *et al.*, 2014
Arg174Ala	<1	Chang *et al.*, 2014
Thr178Ala	<1	Chang *et al.*, 2014
Arg261Ala	<1	Chang *et al.*, 2014
Glu21Ala	1.4	Manat *et al.*, 2015
Ser27Ala	0.013	Manat *et al.*, 2015
Ser173Ala	2.2	Manat *et al.*, 2015
Arg174Ala	0.13	Manat *et al.*, 2015

*Relative specific activity refers to the enzymatic activity of the BacA *E. coli* construct as a percentage of wild type. Specific activity measurements were made with C55PP as substrate in the current and the Chang *et al.*, (2014)[3] studies. In the Manat *et al.*, (2015)[4] work, C15PP was used as substrate. Only mutations that reduced specific activity to <3 % of wild type values are included from the Chang *et al.*, (2014) and Manat *et al.*, (2015) studies.

Supplementary Table 2 | Primers used for BacA site directed mutagenesis.

Primers	Sequence (5' to 3')
bacA G42C-f	CACTTGTTGGGGTTTGAGTGCGACACGGCGAAAACCTTT
bacA G42C-r	AAAGGTTTTCGCCGTGTCGCACTCAAACCCCAACAAGTG
bacA F117C-f	GACACGATTAAGTCATTGTGTAACCCGATAAATGTGATG
bacA F117C-r	CATCACATTTATCGGGTTACACAATGACTTAATCGTGTC

Supplementary References

1. Crooks, G. E., Hon, G., Chandonia, J. M. & Brenner, S. E. WebLogo: a sequence logo generator. *Genome Res*. **14**, 1188-1190 (2004).

2. Ovchinnikov, S. *et al*. Large-scale determination of previously unsolved protein structures using evolutionary information. *Elife* **4**, e09248 (2015).

3. Chang, H. Y., Chou, C. C., Hsu, M. F. & Wang, A. H. Proposed carrier lipid-binding site of undecaprenyl pyrophosphate phosphatase from *Escherichia coli*. *J*. *Biol*. *Chem*. **289**, 18719-18735 (2014).

4. Manat, G. *et al*. Membrane topology and biochemical characterization of the *Escherichia coli* BacA undecaprenyl-pyrophosphate phosphatase. *PLoS One* **10**, e0142870 (2015).
