# Supplementary material for: Crystal structure of undecaprenyl-pyrophosphate phosphatase and its role in peptidoglycan biosynthesis
Source: Nat Commun. 2018 Mar 14;9:1078. doi: 10.1038/s41467-018-03477-5 (PMC5852022; doi:10.1038/s41467-018-03477-5)
Supplement: Supplementary file 3 — Description of Additional Supplementary Information [file 41467_2018_3477_MOESM3_ESM.docx]

**Description of Additional Supplementary Files**

File Name: Supplementary Movie 1

Description:

Supplementary Movie 1 | MDS of steps in the undecaprenyl-pyrophosphate phosphatase reaction showing the full dynamics of the enzyme. The movie shows the molecular simulations of the states described in Extended Data Figure 10. The order of the simulations is as follows: (a) Resting state, with Ser27 protonated, Glu21 deprotonated and Arg174. (b) Binding of C55PP in the active site. (c) Formation of the Ser27-C55PP pentavalent intermediate. (d) Reaction product C55P with phosphorylated Ser27. (e) C55P exits the binding site leaving the phosphorylated Ser27. Upon release of the phosphate from and reprotonation of Ser27 the enzyme returns to its original resting state (a).

File Name: Supplementary Movie 2

Description:

Supplementary Movie 2 | MDS of steps in the undecaprenyl-pyrophosphate phosphatase reaction with loops, H8 and much of the polyisoprenyl tail of C55P and C55PP removed for clarity. The movie shows the molecular simulations of the states described in Extended Data Figure 10 and Extended Data Movie 1A. The order of the simulations is as follows: (a) Resting state, with Ser27 protonated, Glu21 deprotonated and Arg174. (b) Binding of C55PP in the active site. (c) Formation of the Ser27-C55PP pentavalent intermediate. (d) Reaction product C55P with phosphorylated Ser27. (e) C55P exits the binding site leaving the phosphorylated Ser27. Upon release of the phosphate from and reprotonation of Ser27 the enzyme returns to its original resting state (a).

File Name: Supplementary Movie 3

Description:

Supplementary Movie 3 | Molecular movie of the interpolation between crystal structure and the alternate, inverted state. The movie shows the transitions between periplasm- and cytoplasm-facing states, viewed in turn from the membrane plane, the periplasmic side, the cytoplasmic side and finally the alternate face of the membrane plane.
